# Association between body mass index and mental health among nurses: a cross-sectional study in China

**DOI:** 10.1186/s12913-024-11006-y

**Published:** 2024-04-24

**Authors:** Bonan Luan, Xueyan Tian, Chao Wang, Ming Cao, Dongmei Liu

**Affiliations:** 1 Department of Operating Room, Shengjing Hospital of China Medical University, Shenyang, P.R. China; 2 Department of Urology, Shengjing Hospital of China Medical University, 36 Sanhao Street, Shenyang, Liaoning 110004 P.R. China

**Keywords:** BMI, Obesity, Depression, Anxiety, COVID-19

## Abstract

**Purpose:**

To examine the correlation between body mass index (BMI) and mental well-being in Chinese nurses during the COVID-19 epidemic.

**Method:**

This study was conducted in a tertiary hospital using a cross-sectional design. A total of 2,811 nurses were enlisted at Shengjing Hospital in China during the period from March to April, 2022. Information was gathered through a questionnaire that individuals completed themselves. The mental health of the participants was assessed using the Patient Health Questionnaire-9 and the Generalized Anxiety Disorder Assessment-7. Binary logistic regression was used to calculate adjusted odds ratios (ORs) and their corresponding 95% confidence intervals.

**Results:**

The prevalence of nurses experiencing depression and anxiety was 7.8% (219) and 6.7% (189), respectively. Regarding depression after adjustment, the odds ratios (ORs) for each quartile, compared to the lowest quartile, were as follows: 0.91 (95% confidence interval [CI]: 0.53, 1.56), 2.28 (95% CI: 0.98, 3.77), and 2.32 (95% CI: 1.41, 3.83). The p-value for trend was found to be 0.001. The odds ratios (ORs) for anxiety after adjustment were 2.39 (0.83, 4.36), 4.46 (0.51, 7.93), and 2.81 (1.56, 5.08) when comparing the highest quartiles to the lowest quartile. The *p*-value for trend was 0.009.

**Conclusion:**

This study found a positive association between BMI and poor mental health among nurses during the COVID-19 pandemic, particularly in those who were overweight or obesity. The findings could assist in developing interventions and help policy-makers establish appropriate strategies to support the mental health of frontline nurses, especially those who are overweight or obesity.

**Supplementary Information:**

The online version contains supplementary material available at 10.1186/s12913-024-11006-y.

## Introduction

Depression and anxiety are the most common mental health illnesses worldwide [1]. Depression is a mood disorder that affects an individual’s thoughts and feelings and leads to persistent feelings of sadness and disinterest [2]. Anxiety is a group of mental disorders characterized by nervousness, apprehension, and fear [3]. Depression and anxiety disorders are major contributors to the mental health burden of adults [4]. Poor mental health often affects regular activities and probably results in poor professional performance. Given the detrimental effects of depression and anxiety on physical and mental health, it is important to explore the relevant factors, and to thereby contribute toward preventing the development of mental health disorders [5].

According to the National Institute of Health of America, body mass index (BMI) is a measure that defines individuals as underweight, normal weight, or overweight, that is calculated using their weight and height [6]. Recent research indicates that high BMI and obesity continue to relentlessly increase globally, with approximately two billion people being overweight or obese [7]. In a meta-analysis including 57 prospective studies and 900,000 adults, they found that above 25 kg/m^2^, positive associations between BMI and cardiovascular disease, hypertension, diabetes mellitus, stroke, and cancer were recorded in both sexes. Moreover, each 5 kg/m^2^ higher BMI was associated with about 30% higher overall mortality [8]. Obesity-related diseases have become the fifth leading cause of death worldwide [9].

A systematic review and meta-analysis on the longitudinal relationship between BMI and mental health, they found that obesity at baseline increased the risk of onset of depression and the unadjusted ORs were 1.55 (including 15 included studies and 58,745 participants) [10]. Moreover, another meta-analysis of 8 Mendelian randomization studies indicated that obesity is a causal risk factor for elevated risk of depression (OR = 1.33) [11]. Previous studies also demonstrate a bi-directional relationship between obesity and mental health [12]. Although these existing studies address the issue of obesity and mental health, none of these studies address this issue among nurses.

As nurses fulfill an essential role among healthcare workers, they experienced a particularly high occupational burden during the peak of coronavirus disease (COVID-19) pandemic [13]. In a multi-center cross-sectional online survey, among 395 healthcare workers, there were 42.28% and 56.2% were found to have depression, and anxiety during the COVID-19 pandemic, respectively [14]. A recent study conducted in 2020 from China shows that nurses experienced more unfavorable mental health outcomes than other healthcare workers during the pandemic [15]. Furthermore, for nurses, poor mental health may influence not only themselves but also their professional performance and the quality of the health care provided, even affecting patient safety [16, 17]. A growing body of evidence suggests that individuals with changes in BMI have experienced deteriorating symptoms, such as isolation, anxiety and depression as a result of the COVID-19 pandemic compared to previous timepoints. The increasing obesity rates may have modestly increased the prevalence of depressive symptoms in the general population [18]. However, there is currently no data to explore the association between BMI and mental health among nurses during the COVID-19 pandemic. To fill this gap, we conducted a large cross-sectional study to explore the association between BMI and mental health among nurses in China during the COVID-19 pandemic.

### Methods

#### Study design

**Fig. 1:**
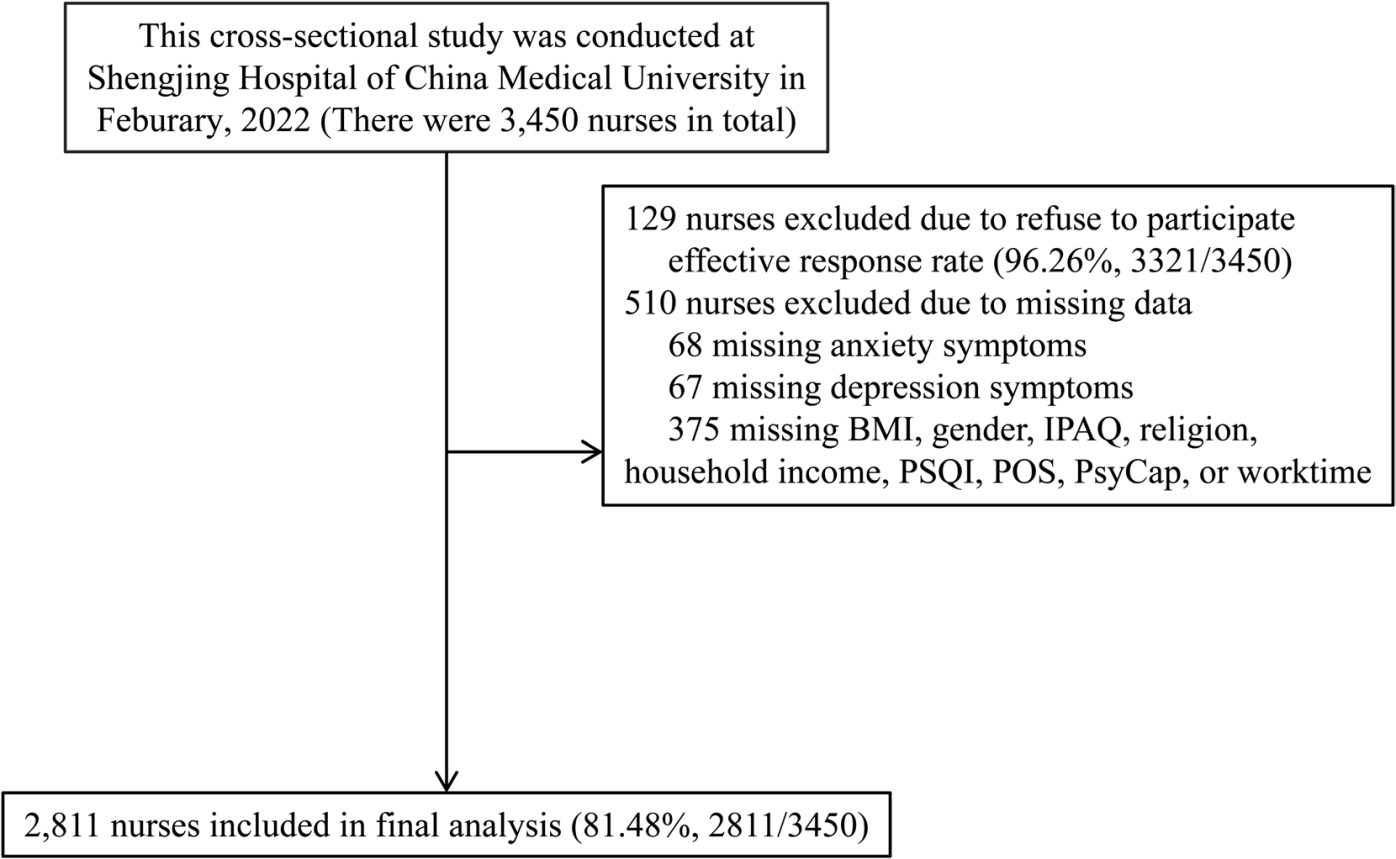
Flowchart of this study

The present cross-sectional investigation was carried out at a Chinese hospital throughout the period from March 2022 to April 2022. The survey was conducted by the nursing department, and it included a total of 3,450 nurses who were employed at the hospital. In the end, a grand total of 2,811 individuals supplied valid and useful responses, leading to an effective response rate of 81.49%. An ensemble of web-based surveys that individuals completed themselves was utilized. Participants successfully filled out a well-organized questionnaire within a time frame of 20 to 25 min. Figure 1 provides a visual representation of the specific information using a flow chart.

The Ethics Committee of Shengjing Hospital Affiliated China Medical University granted ethical permission (2022PS753K). All participants provided written informed consent. The procedures were carried out in accordance with the ethical guidelines outlined in the 1975 Declaration of Helsinki.

### Inclusion and exclusion criteria

The criteria for inclusion were as follows: nurses who were currently employed in hospitals and actively working. The study employed the following exclusion criteria: nurses who had engaged in employment for less than three months or had not completed the psychological questionnaire in its whole were disqualified.

### Measurement of covariates characteristics

This study gathered data from the nursing staff on various aspects, including demographic characteristics, dietary habits, life-related factors, work-related factors, experienced important life events, history of physical sickness, exposure to the COVID-19 pandemic, and psychological assessments.

The demographic data encompassed age, gender, and body mass index (BMI), which was self-reported by the participants and measured in kg/m^2^. The individual's dietary habits encompassed their smoking status, alcohol consumption patterns, and coffee consumption patterns. Smoking behavior was classified into three categories: current smokers (those who smoked at least one cigarette per day and had done so for at least six months), former smokers (those who had stopped smoking for at least six months), and non-smokers. Alcohol and coffee consumption patterns are classified into three categories: current drinkers (those who use alcohol or coffee at least once a day and have been doing so for at least six months), former drinkers (those who have stopped consuming alcohol or coffee for at least six months), and non-drinkers (those who do not consume alcohol or coffee).

Life-related factors encompassed various aspects such as sleep quality (measured by PSQI, Pittsburgh Sleep Quality Index scores), physical activity (assessed using IPAQ, International Physical Activity Questionnaire, in terms of Mets × hour/week), religious affiliation, marital status, presence of siblings, monthly household income (in RMB, yuan), occurrence of major life events, history of chronic disease, and frequency of visiting friends.The researchers evaluated the level of physical activity (PA) in the past week using the abbreviated version of the International Physical Activity Questionnaire [19]. The Pittsburgh sleep quality index (PSQI) [20] was used to assess sleep quality.

Work-related variables including employment, field of expertise, weekly working hours, and night shifts. Exposure to the COVID-19 pandemic pertains to nurses who may come into touch with patients suspected or confirmed to have COVID-19, or find themselves in a situation that necessitates COVID-19 quarantine.

### Measurements of psychological variates

The level of organization support was measured using the Chinese version of the Perceived Organization Support Questionnaire (POS) [21]. The Cronbach's α coefficient for POS was 0.921. The Chinese version of the 24-item Psychological Capital Questionnaire (PCQ) [22, 23] was used to assess PsyCap. The Cronbach's α coefficients for self-efficacy, hope, resilience, and optimism were 0.921, 0.936, 0.920, and 0.900, respectively.

### Measurement of depression and anxiety

The assessment of depressive symptoms was conducted using clinically validated measures, specifically the PHQ09 [24]. The PHQ09 scale consists of nine items, each with a 4-point Likert-type scale answer. These responses indicate the frequency of individuals' feelings during the preceding two weeks, ranging from 0 to 3. The cumulative score spans from 0 to 27, with a higher value denoting a greater intensity of depression symptoms. A PHQ09 score of 10 or more was used to determine the presence of serious depression.

The Chinese version of the GAD07 [25] was used to assess anxiety symptoms. The GAD07 questionnaire comprises 7 items, with each item being responded to on a 4-point Likert-type scale ranging from 0 (indicating never) to 3 (indicating always). A greater score indicates a higher level of anxiety symptoms. A GAD07 standardized score of 10 or higher was used to characterize the presence of significant anxiety symptoms. The Cronbach's α coefficients for the Patient Health Questionnaire-9 (PHQ-9) and Generalized Anxiety Disorder-7 (GAD-7) were 0.951 and 0.928, respectively.

### Sample size calculation

The confidence level (1-α) was 0.95; the proportion of main outcome (depression and anxiety) was 0.1; The confidence interval width (two sided) was 0.03. The confidence interval formula was Exact (Clopper-Pearson); the 2-tailed P value was 0.05. The sample size was 1,603. It was calculated by PASS 11.0 (Power Analysis and Sample Size 11.0, NCSS Inc., USA) [8, 10, 18].

### Statistical analysis

The data were analyzed using SPSS 22.0 for Windows, developed by SPSS Inc. in Chicago, IL, USA. The continuous variables were reported as the median together with the interquartile range. The categorical variables were presented as the count (proportion). Discrete sets of data that are neither related or dependent on each other. The mean of two continuous variables that follow a normal distribution was compared using the Student's t-test. The Mann–Whitney U test was employed to compare the average values of two continuous variables that do not follow a normal distribution. On the other hand, the χ2 test or Fisher's exact test were utilized for categorical variables.

The quartiles were determined by categorizing the BMI values of all participants depending on their distribution, and these quartiles were then utilized for subsequent research. The study investigated the association between quartile categories of BMI and the presence of poor mental health, specifically depression and anxiety, using binary unconditional logistic regression analysis. The dependent variable in this study was the individual's mental health state, whereas the independent variable was their BMI. The crude odds ratio (OR) was calculated using crude data, and model 1 was further modified for age and gender. Model 2 further accounted for baseline variables that were deemed clinically significant or had a *p*-value < 0.10 in the univariate analysis. These variables included alcohol consumption, sleep quality, number of siblings, experience of major life events, frequency of visiting friends, years of employment, duration of work hours, psychological characteristics related to depression, age, physical activity, marital status, history of chronic disease, specialty, and psychological characteristics related to anxiety. The Model 3 was modified to account for all baseline variables. Adjusted OR and their corresponding 95% confidence intervals (95% CI) were calculated using binary unconditional logistic regression, taking into account any confounding factors. The study examined the presence of a linear trend by analyzing the median value of each quartile as a continuous variable. All *P* values were calculated using a two-tailed test, and the observed difference was considered statistically significant when the *P* value was less than 0.05.

## Results

A total of 2,811 nurses were ultimately enrolled in the study, with a median age of 35 years and a median BMI of 21.83 kg/m^2^. Female participants constituted the majority (94.20%). Out of the total, 69.9% (1,965) of the nurses had a normal weight, 6.3% (177) were underweight, 19.9% (558) were overweight, and 3.9% (111) were obese. The occurrence of depression and anxiety among nurses was 7.8% (219 out of 2,811) and 6.7% (189 out of 2,811), respectively; see details in Table 1 (the distribution of characters by outcome status) and supplementary Table 1**(**the distribution of characters by BMI status**)**.

**Table 1:** Baseline characteristics according to mental health in the cohort analysis (*n* = 2,811)

	**Total**	**Depression**			**Anxiety**		
**Variables**		**Depression**	**Without depression**	***p***	**Anxiety**	**Without anxiety**	***p***
**Number** (%)	2,811	219 (7.80)	2,592 (92.20)		189 (6.70)	2,622 (93.30)	
**Demographic characteristics**							
Age (years)	35.00 (32.00,37.00)	35.00 (32.00, 37.00)	35.00 (32.00, 37.00)	0.810	35.00 (33.00, 38.00)	35.00 (32.00, 37.00)	< 0.001
Gender (female)	2,649 (94.20)	210 (95.90)	2,439 (94.10)	0.277	180 (95.20)	2,469 (94.20)	0.542
BMI (kg/m^2^)	21.83 (20.07,23.88)	22.57 (20.76, 24.38)	21.76 (19.96, 23.80)	0.002	22.66 (20.76, 24.09)	21.77 (19.95, 23.83)	0.005
**Dietary habits**							
Smoking habit				0.659			0.431
Current	36 (1.30)	3 (1.40)	33 (1.30)		3 (1.60)	33 (1.30)	
Former	24 (0.90)	3 (1.40)	21 (0.80)		3 (1.60)	21 (0.80)	
Never	2,751 (97.90)	231 (97.30)	2,538 (97.90)		183 (96.80)	2,568 (97.90)	
Alcohol habit				0.039			0.624
Current	192 (6.80)	24 (11.00)	168 (6.50)		15 (7.90)	177 (6.80)	
Former	144 (5.10)	12 (5.10)	144 (5.40)		9 (4.80)	135 ( 5.10)	
Never	2,475 (88.00)	198 (83.50)	2,355 (88.00)		165 (88.10)	2,310 (88.10)	
Coffee habit				0.606			0.621
Current	768 (27.30)	60 (27.40)	708 (27.30)		45 (23.80)	723 (27.60)	
Former	423 (15.00)	39 (17.80)	384 (14.80)		36 (19.00)	387 (14.80)	
Never	1,620 (57.60)	120 (54.80)	1,500 (57.90)		108 (57.10)	1,512 (57.70)	
**Life related factors**							
Sleep quality (PSQI scores)	5.00 (3.00,8.00)	10.00 (8.00, 12.00)	5.00 (3.00, 7.00)	< 0.001	10.00 (7.00, 12.00)	5.00 (3.00, 7.00)	< 0.001
Physical activity (IPAQ Mets × hour/week)	18.60 (3.65,52.80)	14.60 (0.00, 46.20)	19.30 (4.00, 53.77)	0.824	20.50 (0.00, 87.10)	18.39 (4.00, 51.30)	0.006
Have religion (yes)	93 (3.30)	90 (3.50)	3 (1.40)	0.107	3 (1.60)	90 (3.40)	0.182
Marital status				0.117			0.099
Single	543 (19.30)	48 (21.90)	495 (19.10)		30 (15.90)	513 (19.60)	
Married/cohabitation	2220 (79.00)	171 (78.10)	2049 (79.10)		153 (81.00)	2,067 (78.80)	
Divorce/separation/widow	48 (1.70)	0 (0.00)	48 (1.90)		6 (3.20)	42 (1.60)	
Have siblings (yes)	2,013 (71.60)	168 (76.70)	1,845 (71.20)	0.082	150 (79.40)	1,863 (71.10)	0.015
Household income (Yuan/month)				0.905			0.258
< 5,000	15 (0.50)	3 (1.40)	12 (0.50)		0 (0.00)	15 (0.60)	
≧5,000, < 10,000	462 (16.40)	33 (15.10)	429 (16.60)		39 (20.60)	423 (16.10)	
≧10,000	2,334 (83.0)	183 (83.60)	2,151 (83.00)		150 (79.40)	2,184 (83.30)	
Experienced major events (yes)	1,443 (51.30)	147 (67.10)	1,296 (50.00)	< 0.001	117 (61.90)	1,326 (50.60)	0.003
History of chronic disease (yes)	522 (18.60)	42 (19.20)	480 (18.50)	0.810	45 (23.80)	477 (18.20)	0.056
Visiting friend constantly (no)	75 (2.70)	12 (5.50)	63 (2.40)	< 0.001	9 (4.80)	66 (2.50)	< 0.001
**Work related factors**							
Years of employment				0.100			0.067
< 5 years	384 (13.70)	18 (8.20)	366 (14.10)		12 (6.30)	372 (14.20)	
5–10 years	1,137 (40.40)	96 (43.80)	1,041 (40.20)		87 (46.00)	1,050 (40.00)	
> 10 years	1,290 (45.90)	105 (47.90)	1,185 (45.70)		90 (47.60)	1,200 (45.80)	
Speciality				0.270			0.004
Surgery	1,209 (43.00)	105 (47.90)	1,104 (42.60)		120 (63.50)	1,089 (41.50)	
Internal medicine and others	321 (11.40)	24 (11.00)	297 (11.50)		18 (9.50)	303 (11.60)	
Obstetrics and Gynecology	342 (12.20)	15 (6.80)	327 (12.60)		6 (3.20)	336 (12.80)	
Pediatrics	255 (9.10)	6 (2.70)	249 (9.60)		3 (1.60)	252 (9.60)	
Others	684 (24.30)	69 (31.50)	615 (23.70)		42 (22.20)	642 (24.50)	
Worktime duration (hours/week)				< 0.001			< 0.001
< 40 h	1,758 (62.50)	63 (28.80)	897 (34.60)		54 (28.60)	906 (34.60)	
40–60 h	960 (34.20)	135 (61.60)	1,623 (62.60)		114 (60.30)	1,644 (62.70)	
> 61 h	93 (3.30)	21 ( 9.60)	72 (2.80)		21 (11.10)	72 (2.70)	
Night shifts (more than 3 times/month)	1,590 (56.60)	135 (61.60)	1,455 (56.10)	0.115	117 (61.90)	1,473 (56.20)	0.126
Exposure to the COVID-19 (yes)	315 (11.20)	30 (13.60)	285 (11.00)	0.135	24 (12.70)	291 (11.10)	0.160
**Psychological characteristics**							
POS scores	51.00 (44.00,57.00)	44.00 (36.00, 50.00)	51.00 (45.00, 57.00)	< 0.001	46.00 (40.00, 51.00)	51.00 (45.00, 57.00)	< 0.001
PsyCap-efficacy (scores)	29.00 (24.00,31.00)	24.00 (21.00,26.00)	30.00 (24.14, 32.75)	< 0.001	24.00 (21.00, 28.00)	30.00 (24.00, 32.00)	< 0.001
PsyCap-hope (scores)	30.00 (24.00,32.00)	23.69 (21.00, 26.00)	30.00 (25.00, 32.00)	< 0.001	24.00 (20.00, 29.00)	30.00 (24.00, 32.00)	< 0.001
PsyCap-resiliency (scores)	27.00 (24.00,31.00)	24.00 (21.51, 27.00)	27.00 (24.00, 31.00)	< 0.001	25.00 (23.00, 27.00)	27.00 (24.00, 31.00)	< 0.001
PsyCap-optimism (scores)	26.00 (23.00,28.00)	23.00 (22.00, 25.00)	26.00 (24.00, 29.00)	< 0.001	23.00 (22.00, 24.00)	26.00 (24.00, 29.00)	< 0.001

Categorical variables were reported as the number (percentage). Independent samples Student's t-test was used to compare the mean of two continuous normally distributed variables, and the Mann–Whitney U test was used to compare the mean of two continuous non-normally distributed variables, The χ2 test or Fisher's exact test was used for categorical variables

*Abbreviations*: *BMI* body mass index, *PSQI* Pittsburgh sleep quality index, *IPAQ* International Physical Activity Questionnaire, *COVID-19* Coronavirus Disease 2019, *POS* Perceived Organization Support, *PsyCap* Psychological Capital

Participants with elevated BMI, impaired sleep quality, and diminished scores in perceived organizational support, efficacy, hope, resiliency, and optimism exhibited an increased likelihood of developing depression, as indicated by the univariate analysis. A greater proportion of individuals with depression exhibited concurrent alcohol consumption, had siblings, encountered significant life events, had infrequent social interactions with friends, had employment tenure exceeding five years, and worked in excess of 40 h per week. Individuals who were older, had a higher BMI, experienced poor sleep quality, engaged in lower levels of weekly physical activity, and had lower scores in perceived organizational support, efficacy, hope, resiliency, and optimism were found to have a higher likelihood of developing anxiety. A greater proportion of individuals with anxiety engaged in marriage or cohabitation, had siblings, experienced significant life events, had a background of chronic illnesses, had infrequent social interactions with friends, had a job history exceeding five years, worked for more than 40 h per week, and were employed in the surgical department. The factors stated above exhibited statistical significance in the univariate analysis, as shown in detail in Table 1.

In order to investigate the correlation between BMI and depression, the BMI was divided into four categories based on quartiles. In comparison to the lowest quartile, the odds ratios (ORs) for the other quartiles were as follows: 0.91 (0.53, 1.56), 2.28 (0.98, 3.77), and 2.32 (1.41, 3.83) after making adjustments. Additionally, the p-value for the trend was found to be 0.001. In relation to anxiety, the odds ratios (ORs) for each quartile were as follows: 2.39 (0.83, 4.36), 4.46 (0.51, 7.93), and 2.81 (1.56, 5.08) after adjusting for other factors. Furthermore, there was a significant trend with a *p*-value of 0.009. Refer to the comprehensive information provided in Table 2. We also did sensitivity analysis by excluding participants who were underweight (BMI < 18.5) and only including participants who exposed to the COVID-19 pandemic, these results were consistent with the main outcome; see details in supplementary Tables 2 and 3.

**Table 2:** Association between BMI and mental health among study participants (*n* = 2,811)

	Quartiles of BMI scores (range, *n* = 2,811)				*P* for trend ^a^
BMI level	Level 1 (15.87–21.07)	Level 2 (21.08–22.83)	Level 3 (22.84–24.99)	Level 4 (25.00–40.09)	
**Depression**					
No. of participants	702	708	723	678	
No. of depression	39	48	63	69	
Crude	Reference	1.24 (0.80, 1.91)^b^	1.62 (1.07, 2.45)	1.93 (1.28, 2.90)	0.002
Adjusted model 1 ^c^	Reference	1.24 (0.80, 1.91)	1.62 (1.07, 2.45)	1.93 (1.28, 2.90)	0.002
Adjusted model 2 ^d^	Reference	0.91 (0.53, 1.56)	2.28 (0.98, 3.77)	2.32 (1.41, 3.83)	**0.001**
Adjusted model 3 ^e^	Reference	0.92 (0.53, 1.95)	2.63 (0.95, 4.47)	2.96 (1.72, 5.06)	**< 0.001**
**Anxiety**					
No. of participants	702	708	723	678	
No. of anxiety	24	48	66	51	
Crude	Reference	2.06 (1.24, 3.39)^b^	2.84 (1.76, 4.58)	2.30 (1.40, 3.38)	0.005
Adjusted model 1 ^c^	Reference	1.96 (1.18, 3.24)	2.67 (1.65, 4.32)	2.05 (1.24, 3.39)	0.021
Adjusted model 2 ^d^	Reference	2.39 (0.83, 4.36)	4.46 (0.51, 7.93)	2.81 (1.56, 5.08)	**0.009**
Adjusted model 3 ^e^	Reference	2.30 (0.86, 4.20)	4.28 (0.91, 7.59)	2.60 (1.43, 4.71)	**0.004**

*Abbreviations*: *BMI* body mass index

^a^ Multiple Logistic regression analysis

^b^ Odd ratio (95% confidence interval) (all such values)

^c^ Adjusted for age, and sex

^d^ Additionally adjusted for included alcohol habit, sleep quality, have siblings, experienced major events, visiting friend constantly, years of employment, work-time duration, psychological characteristics for depression; age, sleep quality, physical activity, marital status, have siblings, experience of major events, history of chronic disease, visiting friend constantly, years of employment,speciality, work-time duration, psychological characteristics for anxiety on Model 1

^e^ Additionally adjusted for all baseline variables

## Discussion

Obesity is a major contributor to morbidity and mortality. However, no existing study has focused on the relationship between BMI and mental health among nurses during COVID-19 pandemic. Therefore, we performed a cross-sectional study on a large population of nurses in China. This study showed a positive association between BMI and poor mental health (anxiety and depression) among Chinese nurses during the COVID-19 pandemic, particularly in those who were overweight or obesity.

In line with this, A systematic review and meta-analysis on the longitudinal relationship between BMI and mental health, they found that obesity at baseline increased the risk of onset of depression and the unadjusted ORs were 1.55 (including 15 included studies and 58,745 participants) [10]. Another population-based cross-sectional study enrolled 4,361 Iranian healthcare staff; their results indicate that abdominal obesity was significantly associated with anxiety among females but not among males. It is worth noting that in the current study, most participants were female. At the same time, no significant association was discovered between abdominal obesity and psychological distress in either gender. There was, however, a weak positive association between BMI and depression [26]. Further, a meta-analysis reviewed 25 prospective studies and provided solid evidence of the link between obesity and depression, indicating a bi-directional relationship between BMI and depression [27]. A possible mechanism is the adoption of an unhealthy lifestyle, such as insufficient physical exercise and unhealthy dietary preferences, possibly leading to obesity [27].

The exact underlying pathophysiological mechanism between being overweight and poor mental health is unknown. It has been shown that immune inflammation disorder plays an essential role in mental health disorders such as depression and anxiety. Moreover, a high BMI status can lead to many pro-inflammatory factors in the peripheral circulation system crossing the blood–brain barrier, subsequently inducing depressive-like behaviors. In such cases, the risk of depression and anxiety gradually increases [28, 29]. The association between obesity and disorders such as depression and anxiety may also be explained by hypothalamic–pituitary–adrenal (HPA) axis disorder, leptin, or microbial mechanisms [30–35]. The obesity might involve HPA-axis dysregulation and HPA-axis dysregulation is also well known to be involved in depression. Through HPA axis dysregulation, obesity might cause development to depression. Leptin play an important role in the signaling pathway of glutamatergic neurons for regulating depression-related behaviors, suggesting a possible association between synaptic depression and behavioral manifestations of depression. Depression is associated with decreased gut microbiota richness and diversity. Fecal microbiota transplantation from depressed patients to microbiota-depleted rats can induce behavioural and physiological features characteristic of depression in the recipient animals, including anhedonia and anxiety-like behaviours, as well as alterations in tryptophan metabolism. This suggests that the gut microbiota may play a causal role in the development of features of depression.

While this study provides interesting insights, it is important to acknowledge its various limitations. First, since this study is cross-sectional, there is a concern for reverse causation, where mental health problems may contribute to increased BMI. Future studies with a longitudinal framework are warranted to address this issue. Second, the data were gathered by self-reported questionnaires, specifically pertaining to measurements such as height and weight. It is important to note that this method may be susceptible to recall bias. In addition, given that the majority of the study sample consists of young women, there is a possibility that they may be tempted to falsely report their height and weight. Therefore, this social desirability bias is another limitation of this study. Third, it is important to note that the GAD-7 and PHQ-9 are screening questionnaires that lack the ability to provide clinical diagnosis. This limitation may have had an impact on the outcomes of our study. However, this study is the first to examine the connection between BMI and mental health in nurses during the COVID-19 epidemic while accounting for several influential factors.

### Supplementary Information

**Supplementary Material 1. **

**Supplementary Material 2. **

**Supplementary Material 3. **

## Data Availability

No datasets were generated or analysed during the current study.
