# Supplementary material for: Association between body mass index and mental health among nurses: a cross-sectional study in China
Source: BMC Health Serv Res. 2024 Apr 24;24:506. doi: 10.1186/s12913-024-11006-y (PMC11040996; doi:10.1186/s12913-024-11006-y)
Supplement: Supplementary file 1 — Supplementary Material 1. [file 12913_2024_11006_MOESM1_ESM.docx]

**Supplementary Table 1**. **Baseline characteristics according to BMI status among study participants (n = 2,811)**

|  |  | **BMI (kg/m^2^) quartiles** | | | |  |
| --- | --- | --- | --- | --- | --- | --- |
| **Variables** | **Total** | Level 1 (15.87-21.07) | Level 2 (21.08-22.83) | Level 3 (22.84-24.99) | Level 4 (25.00-40.09) | **p** |
| No. of participants | 2,811 | 702 | 708 | 723 | 678 |  |
| No. of depression | 219 (7.8) | 39 (5.6) | 48 (6.8) | 63 (8.7) | 69 (10.2) | 0.007 |
| No. of anxiety | 189 (6.7) | 24 (3.4) | 48 (6.8) | 66 (9.1) | 51 (7.5) | < 0.001 |
| **Demographic characteristics** |  |  |  |  |  |  |
| Age (years) | 35 (32,37) | 33 (30, 36) | 35 (32, 36) | 35 (32, 37) | 35 (33, 38) | < 0.001 |
| Gender (female) | 2,649 (94.2) | 687 (97.9) | 693 (97.9) | 675 (93.4) | 594 (87.6) | < 0.001 |
| **Dietary habits** |  |  |  |  |  |  |
| Smoking habit |  |  |  |  |  | < 0.001 |
| Current | 36 (1.3) | 9 (1.3) | 0 (0.0) | 15 (2.1) | 12 (1.8) |  |
| Former | 24 (0.9) | 0 (0.0) | 3 (0.4) | 3 (0.4) | 18 (2.7) |  |
| Never | 2,751 (97.9) | 693 (98.7) | 705 (99.6) | 705 (97.5) | 648 (95.6) |  |
| Alcohol habit |  |  |  |  |  | < 0.001 |
| Current | 192 (6.8) | 36 (5.1) | 39 (5.5) | 54 (7.5) | 63 (9.3) |  |
| Former | 144 (5.1) | 24 (3.4) | 15 (2.1) | 39 (5.4) | 66 ( 9.7) |  |
| Never | 2,475 (88.0) | 642 (91.5) | 654 (92.4) | 630 (87.1) | 549 (81.0) |  |
| Coffee habit |  |  |  |  |  | 0.037 |
| Current | 768 (27.3) | 210 (29.9) | 183 (25.8) | 189 (26.1) | 186 (27.4) |  |
| Former | 423 (15.0) | 105 (15.0) | 87 (12.3) | 111 (15.4) | 120 ( 17.7) |  |
| Never | 1,620 (57.6) | 387 (55.1) | 438 (61.9) | 423 (58.5) | 372 (54.9) |  |
| **Life related factors** |  |  |  |  |  |  |
| Sleep quality (PSQI scores) | 5.00 (3.00,8.00) | 5.00 (3.00, 8.00) | 6.00 (3.00, 8.00) | 5.00 (3.00, 7.00) | 5.00 (3.00, 8.00) | 0.063 |
| Physical activity  (IPAQ Mets×hour/week) | 18.60 (3.65,52.80) | 19.80 (4.95, 61.65) | 16.58 (1.85, 49.50) | 16.50 (4.00, 49.25) | 24.13 (6.60, 54.60) | 0.013 |
| Have religion (yes) | 93 (3.3) | 15 (2.1) | 15 (2.1) | 27 (3.7) | 36 (5.3) | 0.0022 |
| Marital status |  |  |  |  |  | < 0.001 |
| Single | 543 (19.3) | 243 (34.6) | 126 (17.8) | 87 (12.0) | 87 (12.8) |  |
| Married/cohabitation | 2220 (79.0) | 441 (62.8) | 573 (80.9) | 621 (85.9) | 585 (86.3) |  |
| divorce/separation/widow | 48 (1.7) | 16 (2.6) | 9 (1.3) | 15 (2.1) | 6 (0.9) |  |
| Have siblings (yes) | 2,013 (71.6) | 384 (54.7) | 540 (76.3) | 558 (77.2) | 531 (78.3) | < 0.001 |
| Household income (Yuan/month) |  |  |  |  |  | 0.001 |
| < 5,000 | 15 (0.5) | 9 (1.3) | 3 (0.4) | 0 (0.0) | 3 (0.4) |  |
| ≧5,000, <10,000 | 462 (16.4) | 96 (13.7) | 138 (19.5) | 105 (14.5) | 123 (18.1) |  |
| ≧10,000 | 2,334 (83.1) | 597 (85.0) | 567 (80.1) | 618 (85.5) | 552 (81.4) |  |
| Experienced major events (yes) | 1,443 (51.3) | 330 (47.0) | 372 (52.5) | 363 (50.2) | 378 (55.8) | 0.010 |
| History of chronic disease (yes) | 522 (18.6) | 102 (14.5) | 144 (20.3) | 117 (16.2) | 159 (76.5) | < 0.001 |
| Visiting friend constantly (no) | 75 (2.7) | 24 (3.4) | 15 (2.1) | 24 (3.3) | 12 (1.8) | 0.039 |
| **Work related factors** |  |  |  |  |  |  |
| Years of employment |  |  |  |  |  | < 0.001 |
| < 5 years | 384 (13.7) | 159 (22.6) | 81 (11.4) | 87 (12.0) | 57 (8.4) |  |
| 5-10 years | 1,137 (40.4) | 300 (42.7) | 270 (38.1) | 312 (43.2) | 255 (37.6) |  |
| > 10 years | 1,290 (45.9) | 243 (34.6) | 357 (50.4) | 324 (44.8) | 366 (54.0) |  |
| Speciality |  |  |  |  |  | < 0.001 |
| Surgery | 1,209 (43.0) | 294 (41.9) | 279 (39.4) | 327 (45.2) | 309 (45.6) |  |
| Internal medicine and others | 321 (11.4) | 96 (13.7) | 84 (11.9) | 69 (9.5) | 72 (10.6) |  |
| Obstetrics and Gynecology | 342 (12.2) | 87 (12.4) | 93 (13.1) | 81 (11.2) | 81 (11.9) |  |
| Pediatrics | 255 (9.1) | 75 (10.7) | 90 (12.7) | 54 (7.5) | 36 (5.3) |  |
| Others | 684 (24.3) | 150 (21.4) | 162 (22.9) | 192 (26.6) | 180 (26.5) |  |
| Worktime duration (hours/week) |  |  |  |  |  | 0.002 |
| < 40 hours | 1,758 (62.5) | 198 (28.2) | 273 (38.6) | 252 (34.9) | 237 (35.0) |  |
| 40-60 hours | 960 (34.2) | 471 (67.7) | 417 (58.9) | 450 (62.2) | 420 (61.9) |  |
| > 61 hours | 93 (3.3) | 33 (4.7) | 18 (2.5) | 21 (2.9) | 21 (3.1) |  |
| Night shifts (more than 3 times/month) | 1,590 (56.6) | 378 (53.8) | 429 (60.6) | 429 (59.3) | 354 (52.2) | 0.003 |
| Exposure to the COVID-19 (yes) | 315 (11.2) | 69 (9.8) | 96 (13.5) | 72 (9.9) | 78 (11.5) | 0.001 |
| **Psychological characteristics** |  |  |  |  |  |  |
| POS scores | 51.00 (44.00,57.00) | 51.00 (43.00, 58.00) | 51.00 (45.00, 57.00) | 51.00 (45.00, 57.00) | 51.00 (44.00, 57.00) | 0.885 |
| PsyCap-efficacy (scores) | 29.00 (24.00,31.00) | 30.00 (24.00,31.00) | 28.00 (24.00, 31.00) | 29.00 (24.00, 33.00) | 30.00 (25.00, 32.00) | 0.023 |
| PsyCap-hope (scores) | 30.00 (24.00,32.00) | 30.00 (24.00, 31.00) | 29.00 (24.00, 31.00) | 30.00 (24.00, 33.00) | 30.00 (25.00, 32.00) | 0.015 |
| PsyCap-resiliency (scores) | 27.00 (24.00,31.00) | 27.00 (24.00, 30.00) | 27.00 (23.14, 30.00) | 27.00 (24.00, 34.00) | 27.00 (25.00, 31.00) | 0.047 |
| PsyCap-optimism (scores) | 26.00 (23.00,28.00) | 26.00 (23.00, 30.00) | 26.00 (23.00, 28.00) | 25.00 (23.00, 27.00) | 25.00 (23.00, 28.00) | 0.037 |

Categorical variables were reported as the number (percentage). One-Way ANOVA test was used to compare the mean of two continuous normally distributed variables, and the Kruskal-Wallis test was used to compare the mean of two continuous non-normally distributed variables, The χ2 test or Fisher's exact test was used for categorical variables.

**Abbreviations**: BMI, body mass index; PSQI, Pittsburgh sleep quality index, IPAQ, International Physical Activity Questionnaire; COVID-19, Coronavirus Disease 2019; POS, Perceived Organization Support; PsyCap, Psychological Capital.
