# Supplementary material for: Association between body mass index and mental health among nurses: a cross-sectional study in China
Source: BMC Health Serv Res. 2024 Apr 24;24:506. doi: 10.1186/s12913-024-11006-y (PMC11040996; doi:10.1186/s12913-024-11006-y)
Supplement: Supplementary file 2 — Supplementary Material 2. [file 12913_2024_11006_MOESM2_ESM.docx]

**Supplementary Table 2. Association between BMI and mental health among study participants excluding underweight (BMI<18.5) (n = 2,442)**

|  | Quartiles of BMI scores (range, n = 2,442) | | | | *P* for trend ^a^ |
| --- | --- | --- | --- | --- | --- |
| BMI level | Level 1 (18.5-21.70) | Level 2 (21.72-23.31) | Level 3 (23.32-25.22) | Level 4 (25.22-40.09) |  |
| **Depression** |  |  |  |  |  |
| No. of participants | 612 | 630 | 591 | 609 |  |
| No. of depression | 21 | 54 | 57 | 57 |  |
| Crude | Reference | 2.64 (1.57, 4.42) ^b^ | 3.00 (1.80, 5.02) | 2.91 (1.74, 4.86) | <0.001 |
| Adjusted model 1 ^c^ | Reference | 2.68 (1.60, 4.49) | 3.06 (1.83, 5.13) | 3.04 (1.81, 5.11) | <0.001 |
| Adjusted model 2 ^d^ | Reference | 2.45 (1.39, 4.34) | 3.50 (1.99, 6.14) | 3.03 (1.73, 5.31) | **<0.001** |
| Adjusted model 3 ^e^ | Reference | 2.72 (1.50, 4.94) | 3.94 (2.19, 7.08) | 4.32 (2.37, 7.85) | **<0.001** |
| **Anxiety** |  |  |  |  |  |
| No. of participants | 612 | 630 | 591 | 609 |  |
| No. of anxiety | 27 | 42 | 60 | 42 |  |
| Crude | Reference | 1.55 (0.94, 2.54) ^b^ | 2.45 (1.53, 3.91) | 1.61 (0.98, 2.64) | 0.019 |
| Adjusted model 1 ^c^ | Reference | 1.59 (0.96, 2.61) | 2.35 (1.47, 3.77) | 1.49 (0.90, 2.45) | 0.055 |
| Adjusted model 2 ^d^ | Reference | 1.12 (0.64, 1.94) | 2.20 (1.31, 3.69) | 2.10 (1.63, 2.90) | **0.009** |
| Adjusted model 3 ^e^ | Reference | 1.20 (0.67, 2.13) | 2.31 (1.36, 3.95) | 2.20 (1.67, 3.20) | **0.008** |

^a^ Multiple Logistic regression analysis.

^b^ Odd ratio (95% confidence interval) (all such values).

^c^ Adjusted for age, and sex.

^d^ Additionally adjusted for included alcohol habit, sleep quality, have siblings, experienced major events, visiting friend constantly, years of employment, work-time duration, psychological characteristics for depression; age, sleep quality, physical activity, marital status, have siblings, experience of major events, history of chronic disease, visiting friend constantly, years of employment,speciality, work-time duration, psychological characteristics for anxiety on Model 1.

^e^ Additionally adjusted for all baseline variables. **Abbreviations:** BMI, body mass index;
