# Supplementary material for: Association between body mass index and mental health among nurses: a cross-sectional study in China
Source: BMC Health Serv Res. 2024 Apr 24;24:506. doi: 10.1186/s12913-024-11006-y (PMC11040996; doi:10.1186/s12913-024-11006-y)
Supplement: Supplementary file 3 — Supplementary Material 3. [file 12913_2024_11006_MOESM3_ESM.docx]

**Supplementary Table 3. Association between BMI and mental health among study participants (only including workers involved in COVID-19) (n = 315)**

|  | Quartiles of BMI scores (range, n = 315) | | | | *P* for trend ^a^ |
| --- | --- | --- | --- | --- | --- |
| BMI level | Level 1 (18.01-21.22) | Level 2 (21.23-22.99) | Level 3 (23.00-25.09) | Level 4 (25.10-34.01) |  |
| **Depression** |  |  |  |  |  |
| No. of participants | 75 | 73 | 89 | 78 |  |
| No. of depression | 4 | 6 | 7 | 9 |  |
| Crude | Reference | 1.59 (0.43, 5.88) ^b^ | 1.52 (0.43, 5.39) | 2.32 (0.68, 7.87) | 0.200 |
| Adjusted model 1 ^c^ | Reference | 1.51 (0.41, 5.19) | 1.45 (0.41, 5.19) | 2.16 (0.61, 7.72) | 0.263 |
| Adjusted model 2 ^d^ | Reference | 1.20 (0.23, 6.16) | 3.35 (0.60, 18.72) | 4.68 (0.82, 26.81) | **0.041** |
| Adjusted model 3 ^e^ | Reference | 1.07 (0.18, 6.32) | 2.98 (0.47, 18.81) | 6.01 (0.87, 41.40) | **0.035** |
| **Anxiety** |  |  |  |  |  |
| No. of participants | 75 | 73 | 89 | 78 |  |
| No. of anxiety | 1 | 4 | 7 | 8 |  |
| Crude | Reference | 4.29 (0.47, 39.33) ^b^ | 6.32 (0.76, 39.33) | 8.46 (0.76, 52.56) | 0.023 |
| Adjusted model 1 ^c^ | Reference | 4.09 (0.44, 37.56) | 6.05 (0.73, 50.56) | 7.83 (0.92, 66.65) | 0.036 |
| Adjusted model 2 ^d^ | Reference | 2.69 (0.21, 35.27) | 7.15 (0.63, 41.36) | 12.23 (1.07, 69.54) | **0.012** |
| Adjusted model 3 ^e^ | Reference | 2.50 (0.16, 39.18) | 6.74 (0.52, 47.13) | 15.85 (1.15, 79.22) | **0.009** |

^a^ Multiple Logistic regression analysis.

^b^ Odd ratio (95% confidence interval) (all such values).

^c^ Adjusted for age, and sex.

^d^ Additionally adjusted for included alcohol habit, sleep quality, have siblings, experienced major events, visiting friend constantly, years of employment, work-time duration, psychological characteristics for depression; age, sleep quality, physical activity, marital status, have siblings, experience of major events, history of chronic disease, visiting friend constantly, years of employment,speciality, work-time duration, psychological characteristics for anxiety on Model 1.

^e^ Additionally adjusted for all baseline variables. **Abbreviations:** BMI, body mass index;
